# Ethical considerations for mystery shopper studies of pharmaceutical sales

**DOI:** 10.2471/BLT.20.250878

**Published:** 2020-06-01

**Authors:** Jack C Collins, Rebekah J Moles, Jonathan Penm, Carl R Schneider

**Affiliations:** a Sydney Pharmacy School, Faculty of Medicine and Health, The University of Sydney, Science Rd, Camperdown, NSW 2006, Australia.

Unsafe use of medicines, such as incorrect drugs or doses and wrong route of administration, is a leading cause of injury and avoidable harm and costs an estimated 42 billion United States dollars annually in health-care costs including hospitalizations. In response, the World Health Organization (WHO) launched in 2017 *Medication Without Harm* as the theme for the third Global Patient Safety Challenge.^1^^–^^3^

One way to assess medication errors is to observe the behaviour of health-care professionals in the safe prescribing and supply of medicines. Administrative data, including medical records and practitioner or patient self-reports and ethnographic approaches, can all be used in studies. These methods come with limitations regarding the validity of the data because of limited details and incomplete or selective reporting, the potential for misreporting, recall bias or the effect of observing participants. To overcome these limitations, a covert form of ethnographic research is the use of simulated patients, also called mystery shoppers or pseudo-patients. Researchers might select simulated patient visits as the preferred study design to avoid the potential influence of being observed and obtain results that reflect what can be considered close to actual practice. In the simulated patient method, individuals pose as patients and access health services without the knowledge of health-care providers. Despite these advantages, this method might be perceived as wasting health-care resources, in contrast to other research methods, such as auditing of administrative data. The simulated patient method has been successfully used to investigate numerous clinical scenarios and settings, including a study of illicit sales of antibiotics in China.^4^

Beyond the general principles of ethical research that researchers should consider when undertaking any study, they must pay attention to several considerations when using the simulated patient method. The first consideration is the process for obtaining informed consent from participants. A consent process framework has been suggested for simulated patient studies to describe the methods of obtaining consent.^5^ Consent processes can vary from obtaining consent from each individual participant,^6^ from an institution on behalf of its employees^7^ or from an overarching body, such as a professional organization,^8^ to obtaining ethical approval for a waiver of consent.^9^ Some researchers have conducted such studies without seeking approval from an ethics committee or institutional review board.^10^ As the consent process moves away from individual consent to consent from organizations, the human and financial resources required for research decrease, which may be considered advantageous. However, lack of individual consent is a threat to the autonomy of participants. Additional legislative requirements to obtain individual consent for audio and/or visual recordings in different jurisdictions may exist. Researchers need to consider the objectives of their simulated patient studies, as well as of any expectations of participants and ethics review boards when selecting the process of obtaining consent.

The second issue is the disclosure of findings and their implications. As the simulated patient method directly observes practice, the potential for reporting of unsafe, illegal or unprofessional practice arises. Researchers need to consider which, if any, of these findings will be disclosed, and to whom they will be reported. The implications of disclosure range from participant dismissal, misconduct or criminal proceedings, to disrepute of the participant’s organization or profession. Considering the implications of non-disclosure is also necessary. For example, does non-disclosure of unsafe practice by a practitioner pose a risk to the general public? Researchers should articulate and make participants aware of the disclosure process if unprofessional or illegal practice is observed during the study, notably whether individual participants will be identifiable in the analysis and dissemination of the results.

A final consideration is the inconsistency in application of ethical frameworks, ethical research processes, and ethical–legal norms around the world by both researchers and ethical review boards. What would be considered ethical research practice in one setting may not be considered as such in another setting. As simulated patient studies have become a common and accepted research design in assessing practitioner behaviour, researchers must consider the ethical principles relating to such studies, including the expectations of participants, colleagues and review boards, and sociocultural and ethical-legal norms in the research setting. Researchers should consider if a method other than patient simulation feasibly answers the research question and seek approval or guidance from external reviewers when developing simulated patient studies to investigate the safe and appropriate supply of medicines. Where institutional review boards are unavailable, researchers should consider alternative avenues of review or consult literature in the respective field for guidance to ensure research is conducted ethically in accordance with national guidance where relevant and with the Declaration of Helsinki.^11^
